# The yield of Limulus amoebocyte lysate (LAL), which is used for endotoxin detection and adjunct diagnosis of invasive fungal infection, was optimized with caffeine collection buffer

**DOI:** 10.3389/fmicb.2025.1554313

**Published:** 2025-03-04

**Authors:** Sophia Zhang, Mengmeng Zhang, Jessica Zhang

**Affiliations:** Westford Academy, Westford, MA, United States

**Keywords:** Limulus amoebocyte lysate (LAL), caffeine buffer, exocytosis, endotoxin, fungal infection diagnosis

## Abstract

**Background:**

Limulus amoebocyte lysate (LAL) is a major component of the Fungal Infection Diagnosis Kit for the adjunct diagnosis of invasive fungal infections. During the previous bleeding season, PBS-collection buffer was found to prevent degranulation during the blood collection procedure. In addition, PBS-derived Limulus Amoebocyte Lysate (LAL) was used in both the chromogenic assay and the turbidimetric test. A similar phenomenon was observed with the caffeine collection buffer. This collection buffer is easy to prepare.

**Methods:**

To further confirm these observations, we used caffeine collection buffer to collect blood from horseshoe crabs. Six crabs were bled per week, for a total of 60 crabs. Blood was collected from each crab via both caffeine collection buffer and 3% NaCl collection buffer. The cell pellets were then resuspended in LAL reagent water (LRW) or 5 mM CaCl_2_. The final LAL activity was tested via chromogenic tests and turbidimetric assay methods.

**Results:**

Caffeine collection buffer prevented degranulation for more than 1 h, and the yield of caffeine-derived LAL was much greater than that of the 3% NaCl solution. Notably, caffeine-derived LAL was found to work in both chromogenic tests and turbidimetric assays. The enzyme characteristics of the LAL were also determined.

**Conclusion:**

Caffeine collection buffer prevents amoebocyte degranulation during blood collection and processing. The activity of caffeine LAL is much greater. Caffeine LAL works in both chromogenic tests and turbidimetric assays.

## 1 Introduction

**Figure 1:**
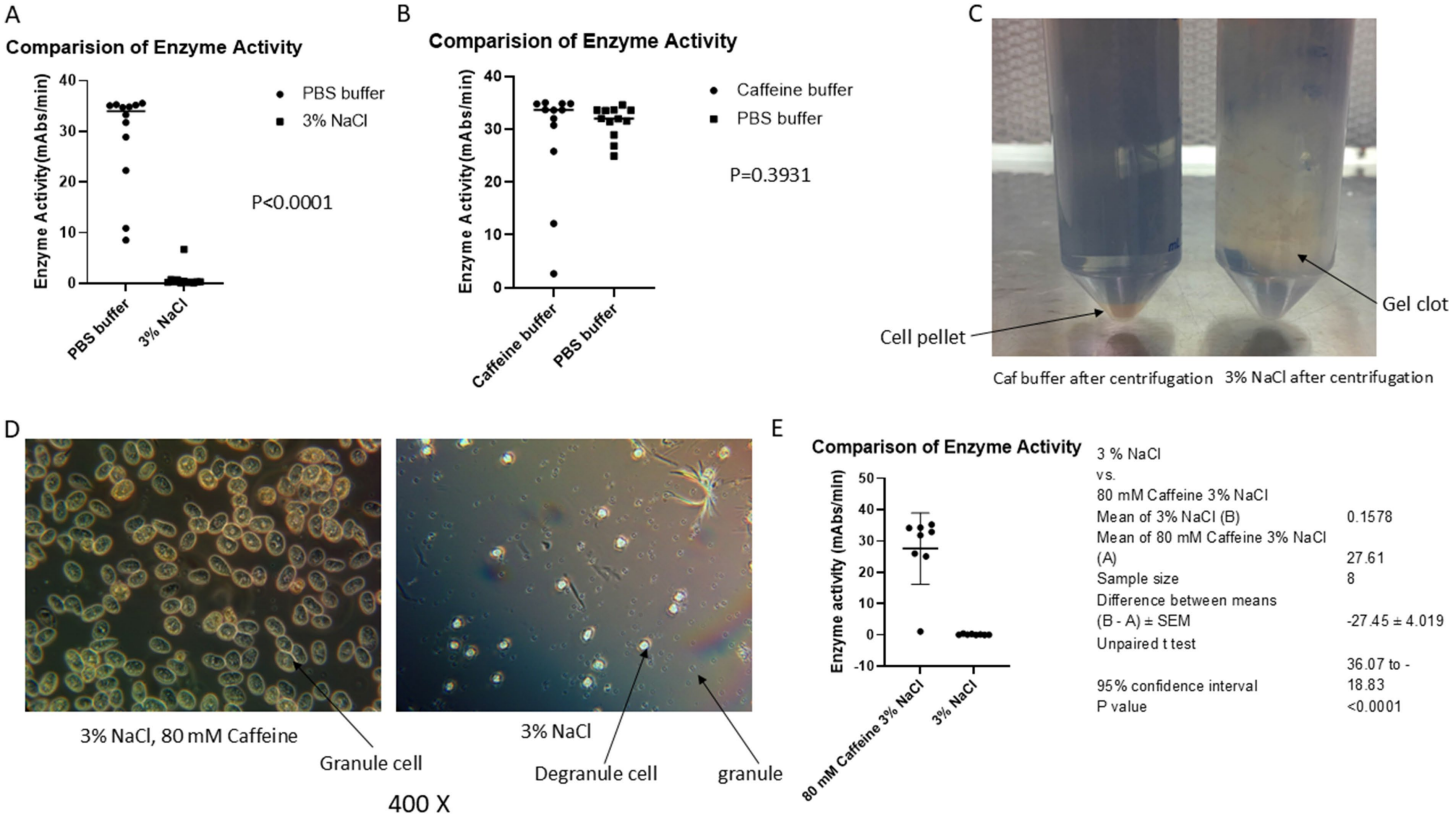
Caffeine buffer inhibits exocytosis during the blood collection process. The blood of horseshoe crabs was collected via caffeine buffer, PBS buffer or 3% NaCl as described in the materials and methods section. **(A)** Comparison of the activity of LAL in PBS with that in 3% NaCl. **(B)** Comparison of the activity of LAL in PBS with that in caffeine. **(C)** After centrifugation, a large gel clot was observed in 3% NaCl solution-collected blood. **(D)** After being kept at 30 min at room temperature, most of the granules were released from 3% NaCl-collected amebocytes; however, almost all the granules were kept inside a cell in a caffeine collection buffer blood mixture under the microscope. **(E)** Comparison of the activity of caffeine LAL with that of 3% NaCl collected LAL. The results revealed that the caffeine-containing enzyme had greater activity (27.61 vs. 0.1578, *p* < 0.0001).

The Limulus amoebocyte lysate (LAL) assay is widely used as an adjunctive test for invasive fungal infection diagnosis in clinics (Tran and Beal, 2016) and endotoxin tests in industry (Gorman, 2020; Górny et al., 1999). LAL is an extract of amoebocytes from the blood of horseshoe crabs. The extraction of LAL is usually composed of three steps: to prepare LAL, raw Limulus amoebocytes are processed from wild-caught *Limulus polyphemus* (“horseshoe crab blood collection-the bleed”); the blood buffer mixture is centrifuged to obtain the amoebocyte pellets; the cell pellet is washed and resuspended in resuspension solution; and LAL is subsequently processed from these amoebocytes. The whole procedure, from blood collection to pellet resuspension into resuspension solution, usually takes more than 1 h. Armstrong and Conrad (2008) described one procedure used to collect blood from American horseshoe crabs in the presence of 3% NaCl. However, this is a lab-based method and is not suitable for large-scale manufacturing processes. Evaluation and experimentation with 3% NaCl collection buffer revealed that Lysis is a misnomer for the discharge of endotoxin-responsive proteins (the LAL cascade) from *Limulus* amoebocytes. This process occurs through a natural degranulation process termed exocytosis, which is consistent with the observation of Solon (Solon et al., 1996). Notably, most granulation fluid was released during the blood collection process when 3% NaCl was used as collection buffer, as shown in our results (Figure 1D). Importantly, degranulation may occur during raw crab blood harvest, during the washing of cell pellets, or during the resuspension of pellets. Throughout collection and washing, the supernatants of the raw blood harvest (hemolymph) and the amoebocyte washing solution are discarded so that any early, degranulation-associated clotting cascade proteins can be lost if degranulation is not well controlled. Previous work has shown that losses can be considerable (81% at steps 1 and 2) (data not shown). Since amoebocyte degranulation is an exocytosis process, preventing the degranulation process from occurring during the blood collection procedure is possible. Introducing degranulation control to the bleeding fluid is beneficial, as it promotes overall granule loss reduction and increases the yield of LAL.

Several methods have been used to control the amoebocyte degranulation process to prevent premature degranulation, which leads to LAL loss before the last step of the resuspension process. The data revealed two processes that positively affected the control of amoebocyte degranulation. The first approach utilized phosphate-buffered saline (pH 6.0), which includes divalent cation chelating agents (EDTA and EGTA), as well as glucose (for cellular energy maintenance, as degranulation is an energy-requiring process) (Diamant and Uvnas, 1961). Finally, the “lysis” or degranulation mixture uses calcium (5 mM CaCl_2_) to increase degranulation. Calcium is utilized in the degranulation medium to restore calcium availability, as degranulation (exocytosis) is an intracellular, calcium wave-dependent phenomenon (Knight et al., 1989).

The data revealed that this approach yielded high levels of LAL activity. LAL preparations made in this manner can be diluted 4-16-fold before the activity stops, as this value is within the range of analytical measurements. The basis of these experiments was the high, endotoxin-concentration activation of serial dilutions of the LAL produced. LAL activity eventually decreased precipitously with serial dilution. Moreover, the greater the LAL cascade component content is, the greater the number of serial dilutions before fall-off. These preparations supported chromogenic and turbidimetric activity.

In addition to the above-described “PBS” method, a second, simpler approach was partially evaluated. The caffeine “bleed method,” which uses a caffeine-rich bleed solution, also appeared to hinder premature degranulation. Experiments were subsequently conducted to evaluate whether a caffeine-based approach utilizing a simpler process might offer similar activity yield benefits. Subsequent observations revealed that a bleed solution composed of 80 mM caffeine in 3% NaCl resulted in degranulation control, and the LAL activity yields were only slightly inferior to those of the PBS-based process. The physiological basis of the blockage of amoebocyte degranulation by caffeine is poorly understood but can be utilized to increase LAL activity yields. Most importantly, caffeine-derived LAL works in chromogenic assays and turbidimetric tests after a 2- to 4-fold dilution.

**Figure 2:**
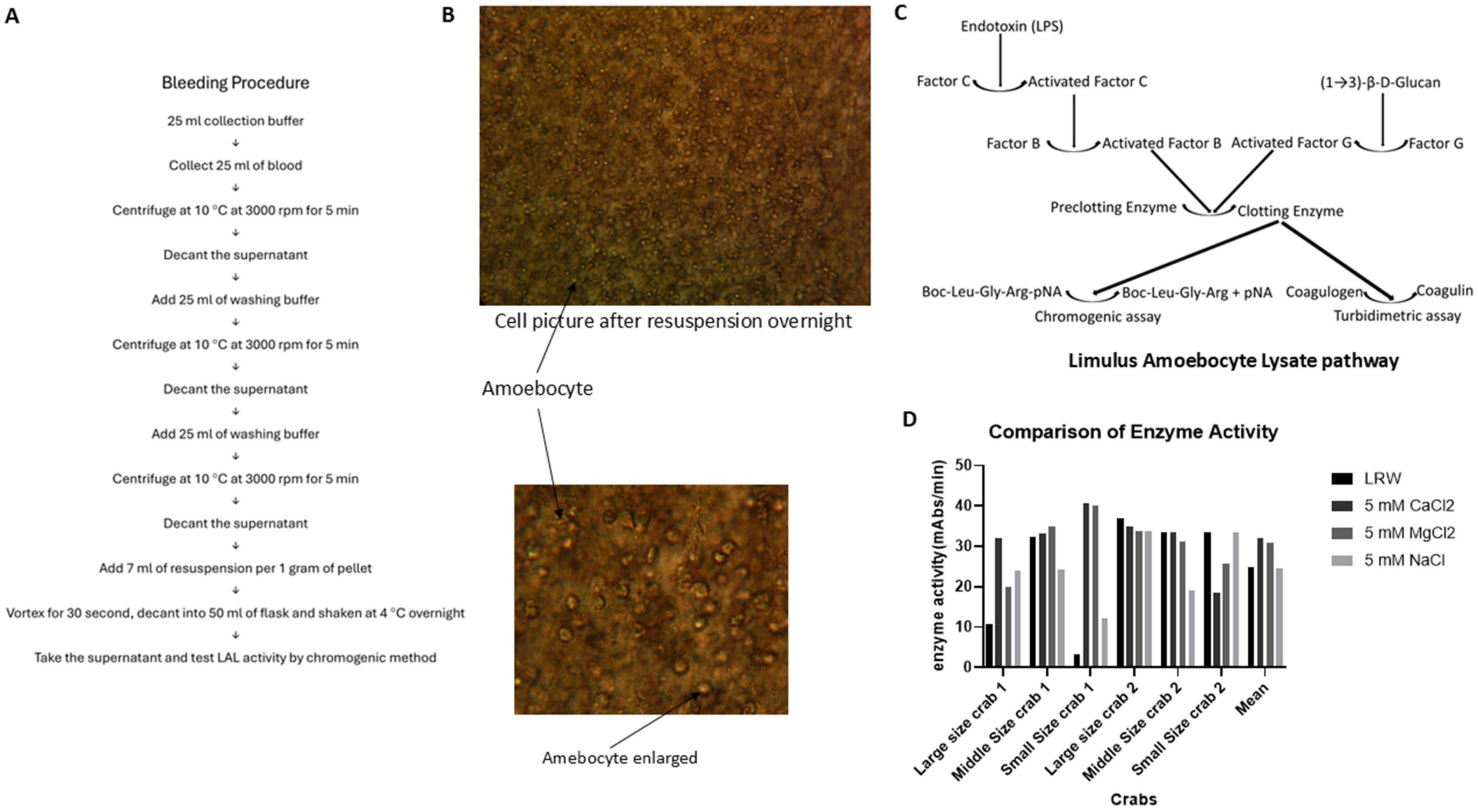
Comparison of LAL enzyme activity in the four different resuspension solutions. A total of 15 mL of blood was collected from one horse-shoe crab in 4 different corn tubes, each containing 15 mL of caffeine buffer. The blood buffer mixture was centrifuged at 3000 rpm at 4°C for 5 min. The cell pellet was washed twice with 3% NaCl. Then, LRW, 5 mM CaCl2, 5 mM MgCl2, and 5 mM NaCl were added to 4 different cell pellets at a ratio of 1 gram of cell pellet per 7 mL of resuspended solution. Afterward, the cell pellet was vortexed for 30 s, the mixture was decanted into 4 different glass flasks, and the flasks were shaken at 4°C overnight. The enzyme activity of the supernatant was tested via a chromogenic method. **(A)** Blood collection procedure. **(B)** The cells remained intact and not lysed after being shaken overnight, with no granules inside the cells. **(C)** Limulus amoebocyte lysate pathway. **(D)** Comparison of enzyme activity in 4 different resuspension solutions.

The chromogenic assay was established via a kinetic photometric method. As shown in Figure 2C, the Limulus lysate contained all the enzymes, such as Factor C, Factor B, Factor G and the preclotting enzyme. The Limulus lysate reacts with both endotoxin and (1,3)-*β*-D-glucan. Both pathways converge on the proclotting enzyme, resulting in its activation and hydrolysis of the chromogenic peptide substrate Boc-Leu-Gly-Arg-*p*-nitroanilide (*p*NA). LAL enzyme activity can be determined by measuring the absorbance of released *p*NA at 405 nm (Iwanaga, 2007). In contrast to the chromogenic assay, the turbidimetric assay is based on the sequential activation of the clotting cascade by a trace amount of endotoxin linked to gelation. Assays were performed in a multiwell tube reader, and turbidity was measured as the optical density (OD) at 660 nm with a multiwell spectrophotometer. The time to obtain the OD threshold value is defined as the onset time. The higher the enzyme activity is, the shorter the onset time is (Iwanaga, 2007; Setlow et al., 1965; Novitsky et al., 1985). Notably, the chromogenic assay uses artificial chromogenic peptide as a substrate of the clotting enzyme, whereas the turbidimetric assay uses the coagulogen in the LAL as a substrate, which is a major component of gelation. The sensitivity of the turbidimetric assay is slightly greater than that of the chromogenic assay. Importantly, the fact that LAL works in chromogenic assays does not mean that it also works in turbidimetric tests. The chromogenic assay requires all the enzymes, such as Factor C, Factor B, Factor G, and preclotting enzymes, to work properly, whereas the turbidimetric assay requires not only that all these enzymes function normally but also that the coagulogen works properly.

This study investigated whether caffeine buffer could block exocytosis and increase the yield of LAL. Sixty crabs were bled, and bleeding occurred once a week during the bleeding season. The crabs used included large, middle, and small crabs. Blood was collected from each crab with both 80 mM caffeine collection buffer (80 mM caffeine, 3% NaCl) and 3% NaCl collection buffer. Afterward, the cell pellets were resuspended in 5 mM CaCl_2_. The final activity of the LAL was tested via both chromogenic and turbidimetric assays.

## 2 Materials and methods

### 2.1 Materials

PBS collection buffer (25 mM KH_2_PO_4_, 25 mM Na_2_HPO_4_, 3% NaCl, 100 mM glucose, 20 mM EGTA, and 10 mM EDTA, pH 6.0), caffeine collection buffer (80 mM caffeine, 3% NaCl, pH 6.0), washing solution (3% NaCl), resuspension solution (5 mM CaCl_2_), and reaction mixture (50 mM MgCl_2_, 1.2 mM chromogenic peptide, and 5 Eu/ml CSE endotoxin) were used.

### 2.2 Bleeding procedure

Twenty-five milliliters of blood were collected in 25 mL of caffeine collection buffer (3% NaCl, 80 mM caffeine). A 20 μL volume of the blood buffer mixture was added to a microslide, which was subsequently covered with a microscope cover glass. Under a microscope, the granule cells and degranulation process were observed. The remaining mixture was centrifuged at 10°C and 3,000 rpm (1,650 × *g*) for 5 min, after which the supernatant was subsequently removed. Then, 25 mL of 3% NaCl washing buffer was added before centrifuging again at 10°C at 3000 rpm (1,650 × *g*) for 5 min, after which the supernatant was removed. Once more, 25 mL of 3% NaCl washing buffer was added, and the mixture was centrifuged at 10°C and 3,000 rpm (1,650 × *g*) for 5 min before the supernatant was removed. At a ratio of 1:7 (1 gram of pellet for every 7 mL of buffer), resuspended buffer was added, and the mixture was vortexed for 30 s. Afterward, the mixture was decanted into 50 mL flasks and shaken overnight at 4°C. The supernatant was tested for LAL activity via the chromogenic method (see Figure 2A).

### 2.3 Chromogenic method test

The activity analysis was performed in 96-well pyro plates. The LAL was diluted 2–16 times with LRW, and 50 μL of LAL was added to the wells in triplicate. Then, 50 μL of the mixture was added (0.14 M Tris, pH 7.4; 50 mM MgCl_2_; 1.2 mM chromogenic peptide; endotoxin 5 Eu/mL). The plate was placed into a BioTek ELx808 plate reader, and the absorbance was read at 405 nm. The reaction was monitored for 60 min, after which Vmean was analyzed and used to compare the enzyme activities of different reaction mixtures (Tran and Beal, 2016; Górny et al., 1999; Ostronoff and Lourenço, 2015).

### 2.4 Lyophilization preparation of the LAL reaction mixture

After the raw LAL was stored at 4°C for 2 weeks, 120 μL of 1 M MgCl_2_ was added to 3 mL of raw LAL. One hundred microliters of 30% NaCl was added, and the mixture was shaken at room temperature for 10 min. The mixture was subsequently centrifuged at 1650 × *g* for 10 min, after which 2.5 mL of the supernatant was transferred to 10 mL sterile glass vials and lyophilized at −50°C for 3 days.

### 2.5 Turbidimetric test

A turbidimetric assay (Sakai et al., 2004; Uchida et al., 2019) was carried out after the LAL was lyophilized at −50°C for 3 days. The tube-based reaction mixtures consisted of 100 μL of a solution containing 160 mM Tris-Cl, pH 7.4, as well as 0.0156, 0.031, 0.062, 0.125, 0.25, 0.5, 1, or 2 EU/mL endotoxin. The lyophilized LAL powder was dissolved in 2.5 mL of 0.2 M Tris-Cl (pH 7.4) buffer at room temperature for 8–10 min before 100 μL of dissolved LAL was added to the reaction mixture; these endotoxin concentrations were halved in the final LAL-containing reaction mixture. The mixture was vortexed for 30 s before being placed in a 96-well PK Flex tube reader. The reaction was subsequently monitored at 660 nm for 180 min.

### 2.6 Statistical analysis

GraphPad Prism 9 was used to perform one-way ANOVA and t tests.

## 3 Results

### 3.1 Caffeine collection buffer inhibits exocytosis during blood collection

Previous data showed that PBS collection buffer prevented the degranulation process during the blood collection procedure, resulting in a greater yield of LAL than 3% NaCl collection solution did (28.86 vs. 0.8794 mAbs/min, *p* < 0.0001; Figure 1A); small-scale experiments indicated that caffeine buffer could also block exocytosis (Figure 1B), although the underlying mechanism was unknown. Notably, the caffeine enzyme activity was slightly lower than that of the PBS enzyme (28.65 vs. 31.39 mAbs/min); however, there was no significant difference (*p* = 0.3931) (Figure 1B). To further confirm the caffeine buffer observations, the blood of 60 crabs was collected via caffeine collection buffer once a week. Six crabs were bled each time, including large (approximately 11–15 pounds), medium-sized (approximately 6–10 pounds), and small (approximately 2–5 pounds) crabs. As shown in Figure 1C, the blood of each crab was collected simultaneously with caffeine collection buffer and a 3% NaCl solution following the procedure in Figure 2A. After the blood buffer mixture was incubated at room temperature for 30 min, the granule cells were observed under a microscope (Figure 1D). The blood mixture was subsequently centrifuged at 3,000 rpm (1,650 × *g*) and 10°C for 5 min. After being washed twice with 3% NaCl, the cell pellets were resuspended in 5 mM CaCl_2_, decanted into 50 mL glass flasks, and shaken at 4°C overnight. The enzyme activity in the flask was tested via a chromogenic method (Iwanaga, 2007). The results indicated that the gel clot formed after 30 min (Figure 1C), and microscopy revealed that many granules were released from the amoebocyte in 3% NaCl solution; however, most of the granules remained inside the amoebocyte when the blood was collected with caffeine buffer (Figure 1D), which is consistent with our macroscopic observations (Figure 1C). The detected enzyme activity was greater in the caffeine-collected LAL; however, almost no enzyme activity was detected in the 3% NaCl-collected LAL (27.61 vs. 0.1578 mAbs/min; *p* < 0.0001) (Figure 1E), which further supported our observations. Notably, caffeine-containing enzymes and sodium chloride-containing enzymes were diluted 4-fold within the range of analytical measurements before the test to observe the difference in enzyme activity. These findings showed that caffeine collection buffers could block granule release during the blood collection process and that the yield of the enzyme activity of LAL was improved.

### 3.2 The addition of 5 mM CaCl_2_ improved the yield of LAL

To test which resuspension solution produced the highest yield of LAL, 4 different corn tubes, each of which contained 15 mL of caffeine collection buffer, were used to collect 15 mL of blood from the same crab. Blood was collected from 2 large crabs, 2 middle crabs, and 2 small crabs. After they were kept at room temperature for 1 h, the blood caffeine collection buffer mixtures were processed following the procedure described in Figure 2A and resuspended in LRW (LAL reagent water), 5 mM CaCl_2_, 5 mM MgCl_2_, or 5 mM NaCl. These cell mixtures were then vortexed for 30 s, shaken at 4°C overnight, and subsequently decanted into 50 mL glass flasks. The overnight cell pellet was observed under a microscope, as shown in Figure 2B. Most of the cells remained intact; additionally, no granules were found inside the cells, indicating that this degranulation process involved exocytosis, not cell lysis. The enzyme activity of the supernatant was tested via chromogenic methods. Notably, this enzyme activity was a cascade of enzyme reactions, as shown in Figure 2C. The enzyme activity varied among the different crabs; the average enzyme activities in the LRW, 5 mM CaCl_2_, 5 mM MgCl_2_, and 5 mM NaCl treatments were 26.08, 35.21, 31.22, and 31.23 mAbs/min, respectively. However, the statistical test revealed that there was no significant difference among the four different resuspension solutions (*p* > 0.05), as shown in Figure 2D. Different crab cells may have different optimal resuspension solutions.

### 3.3 Ca^2+^ inhibits LAL enzyme activity

**Figure 3:**
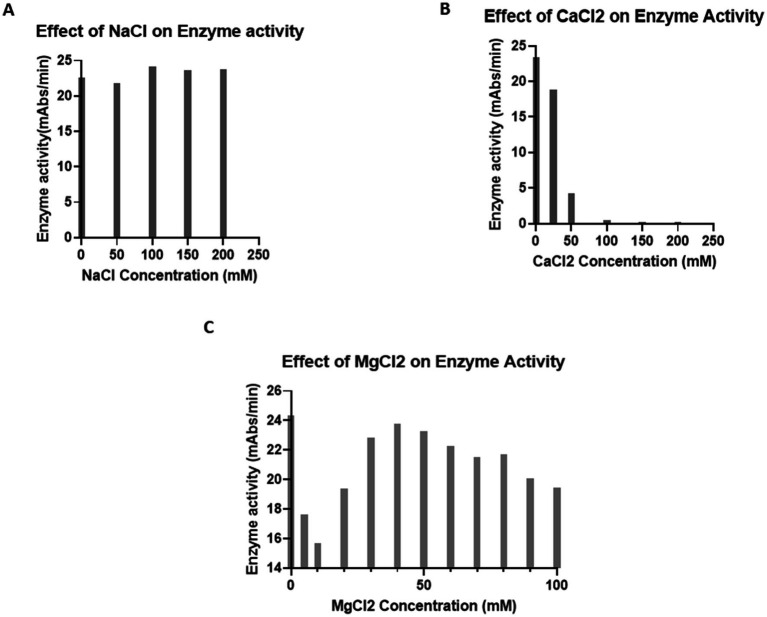
Effect of ions on enzyme activity. **(A)** Effect of NaCl on enzyme activity. A total of 25 μL of LRW-collected LAL was incubated with 0, 50, 100, 150, or 200 mM NaCl in a 96-well plate for 5 min. Afterwards, 50 μL of the reaction mixture was added. The plate was placed in the plate reader, and any reactions were monitored for 1 h. The results showed that NaCl does not inhibit LAL activity. **(B)** Effect of CaCl_2_ on enzyme activity. A total of 25 μL of LRW-collected LAL was incubated with 0, 50, 100, 150, or 200 mM CaCl_2_ in a 96-well plate for 5 min. Afterwards, 50 μL of the reaction mixture was added. The plate was placed in the plate reader, and any reactions were monitored for 1 h. The results indicated that CaCl_2_ inhibited LAL activity. **(C)** Effect of MgCl_2_ on enzyme activity. Forty microliters of LRW-collected LAL were incubated with 0, 5, 10, 20, 30, 40, 50, 60, 70, 80, 90, or 100 mM MgCl_2_ in a 96-well plate for 5 min. Then, 50 μL of the reaction mixture (0.14 M Tris, pH 7.4; 1.2 mM chromogenic peptide; and 5Eu/ml endotoxin) was added. The plate was placed in the plate reader, and the reactions were monitored for 1 h. The results showed that LAL has activity over a broad range of concentrations of MgCl_2_. The optimum concentration of MgCl_2_ was 40 mM.

To test the effects of ions on LAL enzyme activity, LRW-collected LAL was incubated with different concentrations of CaCl_2_, NaCl, and MgCl_2_ in 96-well plates for 5 min. Afterwards, 50 μL of the reaction mixture was added. The plate was placed in the plate reader and monitored for 1 h. NaCl did not affect LAL enzyme activity (Figure 3A); however, CaCl_2_ inhibited LAL enzyme activity (Figure 3B), and more than 80% of the enzyme activity was blocked when the concentration of CaCl_2_ was 50 mM. Notably, LAL enzyme activity was observed over a broad range of MgCl_2_ concentrations (Figure 3C). The optimal concentration of MgCl_2_ ranges from 30 to 50 mM. Interestingly, the LAL examined had heightened enzyme activity, even without MgCl_2_. Some MgCl_2_ from the cell may have contributed to this biochemical reaction.

### 3.4 Effects of pH and temperature on enzyme activity

**Figure 4:**
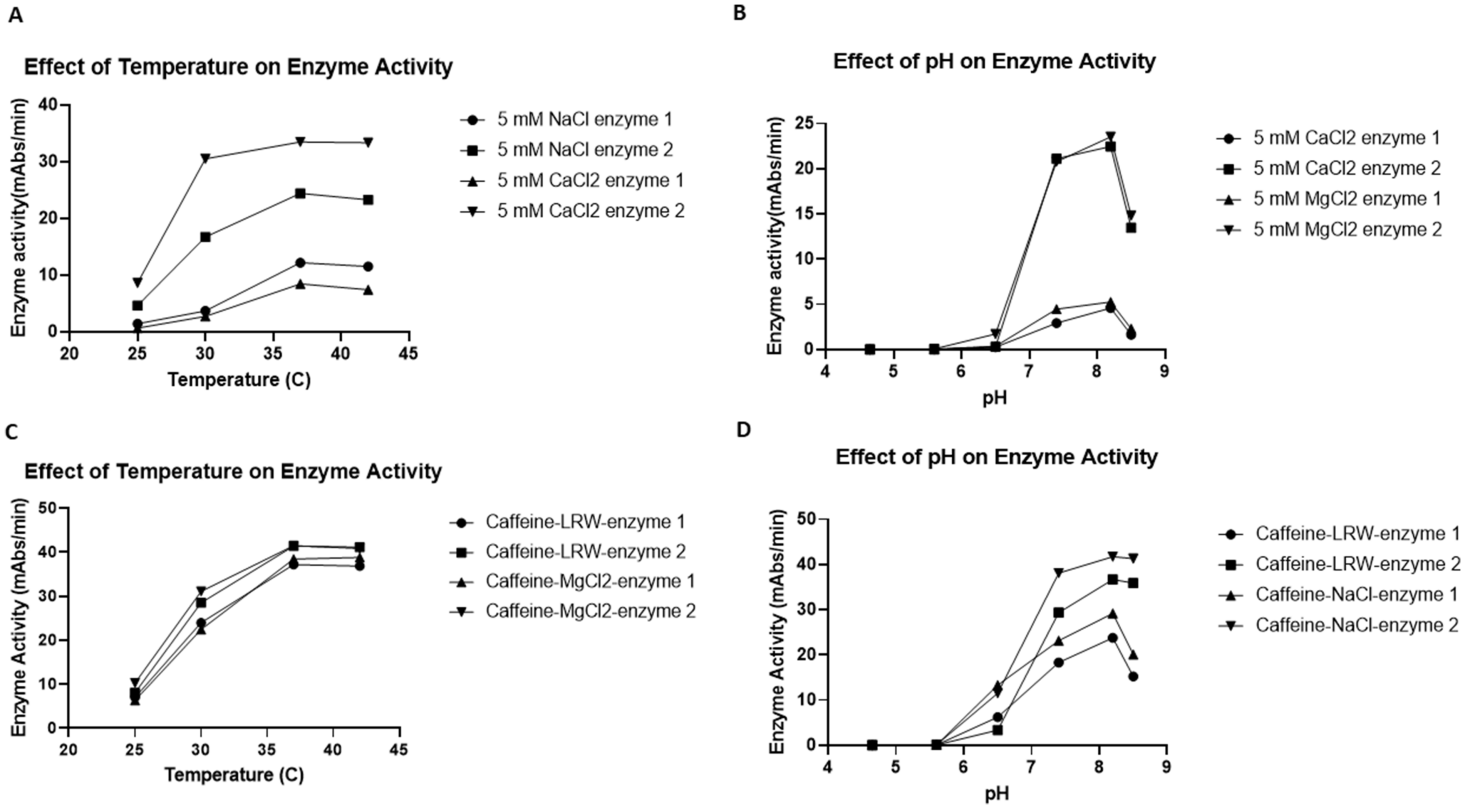
Effects of temperature **(A,C)** and pH **(B,D)** on enzyme activity. **(A,C)** Effects of temperature on enzyme activity. Fifty microliters of the reaction mixture were incubated with 50 μL of LAL in 4 different 96-well plates. The plates were put into four plate readers, which were set at 25°C, 30°C, 37°C and 42°C. The biochemical reaction was monitored for 1 h. The results revealed that the optimum temperature for the LAL enzyme reaction was 37°C. **(B,D)** Effects of pH on enzyme activity. A total of 25 μL of six different pH buffers (200 mM pH 4.6 acetate buffer, 200 mM pH 5.6 acetate buffer, 200 mM pH 6.5 MES buffer, 200 mM pH 7.4 Tris-Cl buffer, 200 mM pH 8.2 Tris-Cl buffer, and 200 mM pH 8.4 Tris-Cl buffer) was incubated with 25 μL of LAL in 96-well plates for 5 min. Then, 50 μL of the reaction mixture (50 mM MgCl_2_, 1.2 mM chromogenic peptide, and 5Eu/mL CSE endotoxin) was added. The plate was placed into the plate reader, and the biochemical reactions were monitored for 1 h. The results indicated that the optimal pH for the LAL enzyme reaction was 8.2.

To test the effect of temperature on enzyme activity, 50 μL of the reaction mixture was incubated with 50 μL of LAL in 4 different 96-well plates. Afterward, the plates were put into four plate readers set at 25°C, 30°C, 37°C, and 42°C. The biochemical reaction was monitored for 1 h. As shown in Figures 4A,C, almost no enzyme activity was detected at 25°C. When the temperature increased from 30°C to 37°C, the enzyme activity gradually increased until it reached its eventual peak before slowly decreasing when the temperature increased from 37°C to 42°C. These findings indicate that the optimal temperature for this enzyme reaction is 37°C (Figures 4A,C).

Commercial LAL test kits such as Fungitell recommend that the optimal pH for the LAL reaction be 7.4. However, the caffeine enzyme was obtained via a different procedure. To test the effect of pH on the caffeine enzyme, 25 μL of buffer with a pH of 4.65, 5.6, 6.5, 7.4, 8.2-, or 8.4 was incubated with 25 μL of LAL in 96-well plates for 5 min. Afterwards, 50 μL of the reaction mixture was added. The plate was placed into the plate reader, and the biochemical reaction was monitored for 1 h. As shown in Figures 4B,D, when the pH of the reaction mixture was less than 6.5, no enzyme activity was detected. From pH values of 7.4 to 8.2, the enzyme activity gradually increased. Since the enzyme activity decreased when the pH reached 8.5, the optimal pH for this caffeine enzyme reaction was assumed to be 8.2. Notably, all eight different enzymes presented similar characteristics in terms of both temperature and pH.

### 3.5 CaCl_2_ inhibited the effect of caffeine collection buffer on exocytosis

**Figure 5:**
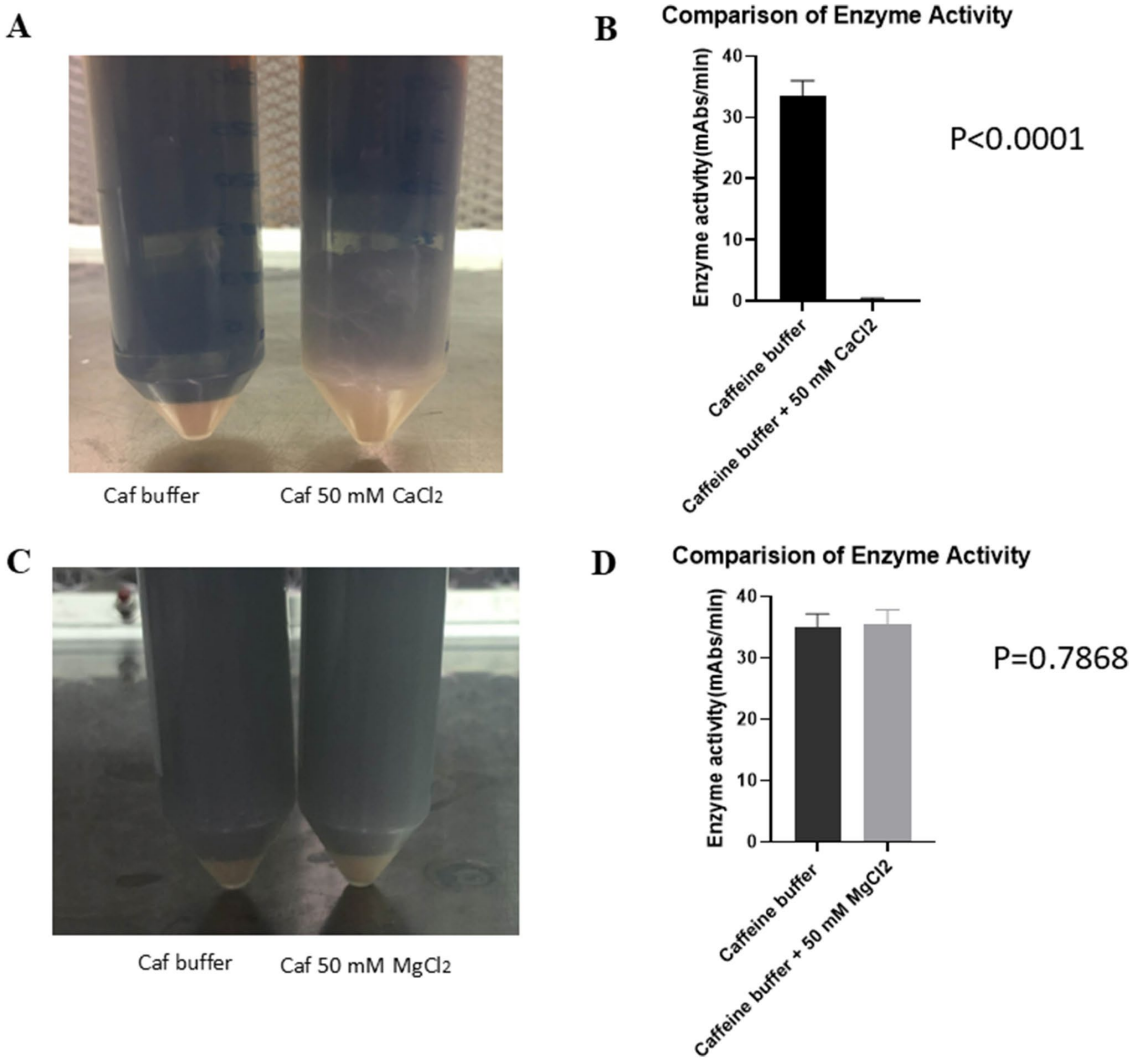
CaCl_2_ inhibited the effect of caffeine buffer on exocytosis. The crab blood was collected using pure caffeine collection buffer and caffeine collection buffer with an additional 50 mM CaCl_2_. **(A)** The gel was formed in caffeine collection buffer with an additional 50 mM CaCl_2_ blood buffer mixture after being incubated at room temperature for 1 h. **(B)** The results of the enzyme activity test revealed that increased enzyme activity was detected for the caffeine-containing enzyme. However, almost no enzyme activity was detected in the supernatant of the caffeine collection buffer supplemented with an additional 50 mM CaCl_2_. **(C)** Crab blood was collected using pure caffeine collection buffer and caffeine collection buffer with an additional 50 mM MgCl_2_. No gel formation was observed in either buffer mixture after being incubated at room temperature for 1 h. **(D)** The results of the enzyme activity test indicated that the enzyme activity in both the pure caffeine collection buffer and the caffeine collection buffer supplemented with 50 mM MgCl_2_ was not significantly different.

The exact mechanism by which caffeine collection buffer blocks exocytosis during blood collection procedures is unknown. To test whether CaCl_2_ could inhibit the effect of caffeine collection buffer on exocytosis, the blood of each crab was collected using caffeine collection buffer and caffeine collection buffer with 50 mM CaCl_2_. The blood buffer mixture was processed as described in the bleeding methods section. Figure 5A shows a gel clot in caffeine collection buffer supplemented with 50 mM CaCl_2_. No similar phenomenon was observed in the pure caffeine collection buffer. Enzyme activity tests revealed that high enzyme activity was detected in the supernatant of the caffeine-collected LAL. However, almost no enzyme activity was detected in the supernatant of the caffeine collection buffer supplemented with 50 mM CaCl_2_ (33.53 vs. 0.1492 mAbs/min, *p* < 0.0001) (Figure 5B), which is consistent with our observations shown in Figure 5A.

A similar procedure was repeated with a caffeine collection buffer and caffeine collection buffer supplemented with 50 mM MgCl_2_. When the blood was collected with caffeine collection buffer supplemented with 50 mM MgCl_2_, no gel formation was observed (Figure 5C). The resulting enzyme activity tests revealed that the enzyme activities in the LAL samples collected with the pure caffeine collection buffer and the LAL samples collected with the caffeine collection buffer supplemented with 50 mM MgCl_2_ were very similar (34.98 vs. 35.52 mAbs/min, *p* = 0.7868) (Figure 5D). These findings suggested that MgCl_2_ could not block the effect of caffeine collection buffer on exocytosis, in contrast to CaCl_2_.

### 3.6 LAL produced by caffeine buffer works in turbidimetric tests

**Figure 6:**
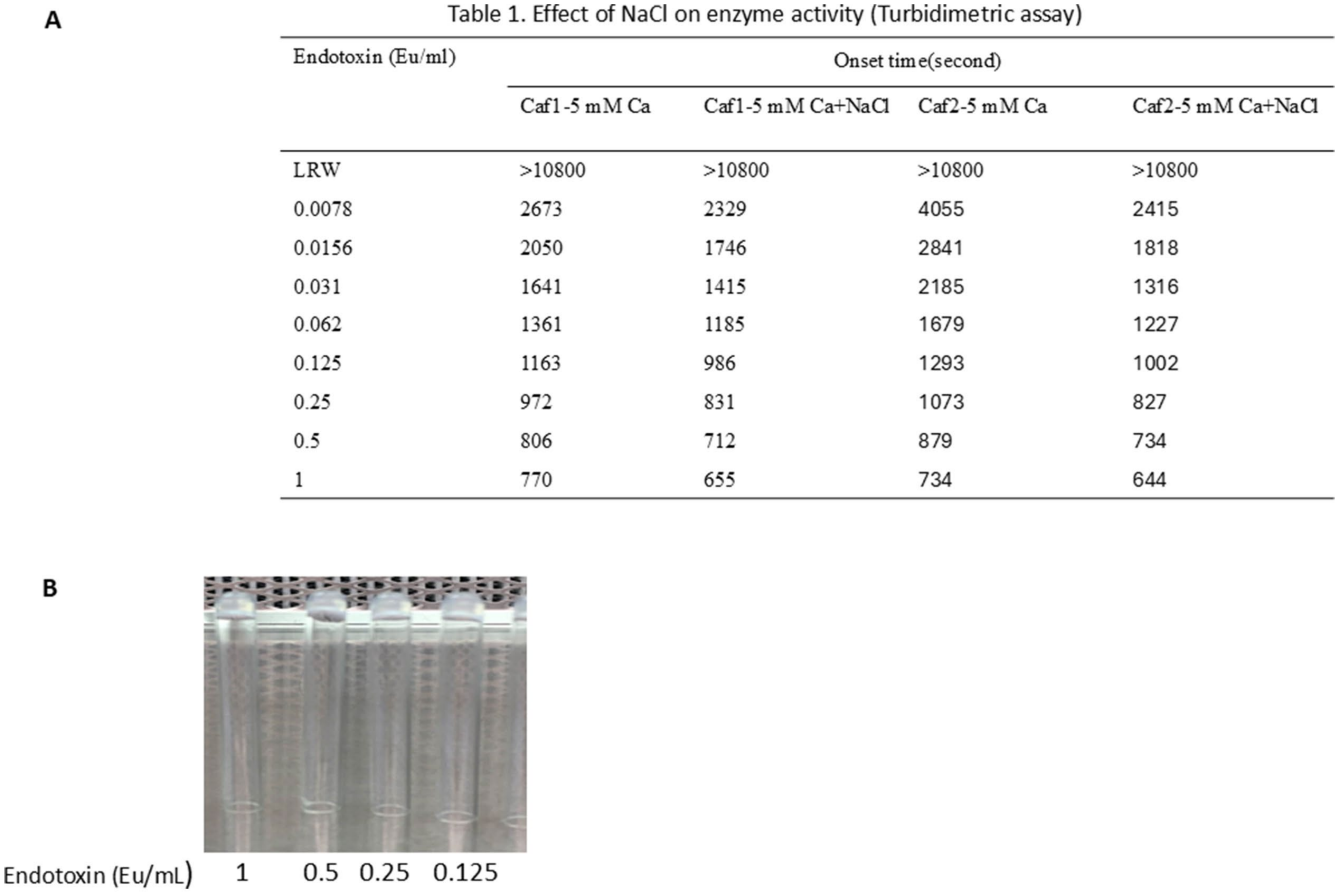
LAL produced through caffeine buffer works in turbidimetric tests. A total of 120 μL of 1 M MgCl_2_ was added to 3 mL of raw LAL in 50 mL glass flasks. One hundred microliters of 30% NaCl was added to one flask, and the same amount of LRW was added to the other. The mixture was shaken at room temperature for 10 min and transferred to 10 mL sterile glass vials for lyophilization at −50°C for 3 days. The tube-based reaction mixtures were composed of 100 μL of a solution containing 160 mM Tris-Cl (pH 7.4) and 0.0156, 0.031, 0.062, 0.125, 0.25, 0.5, 1, or 2 EU/ml endotoxin. The lyophilized LAL powder was dissolved in 2.5 mL of 0.2 M Tris-Cl, pH 7.4, at room temperature for 8 to 10 min. Then, 100 μL of dissolved LAL was added to the reaction mixture. The mixture was vortexed for 30 s before being placed in a 96-well PK Flex plate. The reaction was monitored at 660 nm for 180 min. **(A)** Caffeine LAL works in turbidimetric tests. NaCl seems to increase enzyme activity. **(B)** A gel formed at high concentrations of endotoxin ranging from 0.125 Eu/mL to 1 Eu/mL.

To test whether the LAL produced by caffeine buffer works in turbidimetric tests, the LAL collected using caffeine buffer was incubated at 4°C for 2 weeks. A total of 120 μL of 1 M MgCl_2_ was added to 3 mL of raw LAL in 50 mL glass flasks. In one flask, 100 μL of 30% NaCl was added, and the same volume of LRW was added to the other flasks. The mixture was shaken at room temperature for 10 min and transferred to 10 mL sterile glass vials for lyophilization at −50°C for 3 days. The tube-based turbidimetric test was performed as described in the Methods section. As shown in Figure 6A, the onset time decreased as the endotoxin concentration increased. NaCl seems to increase enzyme activity. A gel clot formed when the endotoxin concentration increased from 0.125 Eu/mL to 1 Eu/mL (Figure 6B). This gel was strong and did not loosen even if the tube was inverted. There seems to be no difference in vision; however, the onset time becomes shorter when the concentration of endotoxin increases. Moreover, when the endotoxin concentration increased, the reaction curve became steeper (data not shown). Notably, the turbidimetric assay does not require artificial chromogenic peptides. This turbidimetric assay properly demonstrated that the functions of all cascade enzymes, such as Factor C, Factor B, preclotting enzyme, and coagulogen, were maintained via the caffeine buffer procedure.

## 4 Discussion

This is the first study in which a caffeine bleeding solution (80 mM caffeine, 3% NaCl) was shown to inhibit exocytosis during blood collection. This approach increases the yield of LAL, a major component of the kit used for endotoxin detection and for adjunctive diagnosis of invasive fungal infection.

The results revealed that up to 80% of the LAL activity was lost when 3% NaCl was used as a crab bleeding solution (Figures 1D,E). However, caffeine collection buffer could control the degranulation process (Figure 1). Solon reported that endotoxin-induced degranulation is an exocytotic process (Solon et al., 1996). Granule release from the cells of *Limulus polyphemus* occurs via exocytosis, not lysis (Figure 2B). These findings suggest that early degranulation (exocytosis) may cause significant activity loss.

To improve our bleeding procedures and increase the yield of LAL activity, we used various degranulation control buffers. The analysis methods included blood collection and processing of cell-free raw LAL, exocytosis inspection by microscopy, and enzyme activity analysis via both chromogenic tests and turbidimetric methods. A variety of low-pH, carboxylic acid-containing bleed solution formulations, such as MSS buffer (citrate, pH 4.6), malic acid buffer (pH 4.2), and lactic acid buffer (pH 4.0), preserve the functions of the enzymatic components of the cascade (data not shown). The divalent cation-chelating ability of carboxylic acids, coupled with the addition of chelators such as EDTA and EGTA, suggest that at least some of the mechanistic bases of degranulation prevention are associated with Ca^2+^ and Mg^2+^ depletion. The subsequent restoration of Ca^2+^ in the final degranulation mixture may benefit this process. The availability of glucose in the initial stages of cell recovery can, in theory, provide the energy needed for the preservation of viability and the energy required for degranulation (Knight et al., 1989).

However, at low pH, organic acid-based buffers harm some components, such as coagulogens, which are required for turbidimetric and gel clot performance. This difference may be due to disulfide reduction and subsequent “scrambling” of the tertiary structure of the coagulin, leading to loss of normal function both as a substrate for the clotting enzyme and for subsequent interactions with the coagulin agent (Kawasaki et al., 2000; Bergner et al., 1996). To preserve the functions of the components, a new higher pH phosphate buffer approach was tested and was found to be successful in preliminary experiments. The pH of the bleeding mixture influences the activity of enzymes. When the pH of the collection buffer was greater than 5.6, the enzyme activity increased (data not shown). However, when the pH of the collection buffer was greater than 6.5, degranulation could occur (data not shown). Therefore, the pH of the collection buffer is a critical variable in designing a successful bleeding solution. Most notably, at a pH greater than 5.6, citric acid, malic acid, or lactic acid buffers could not control degranulation (microscopic observations, data not shown).

To further investigate the potential superiority of this approach, acetic acid buffer and PBS buffer were chosen as the basis for the amoebocyte collection buffers. EGTA and EDTA were added to bind Ca^2+^ and Mg^2+^, respectively. The results showed that the use of PBS was slightly better than the use of acetate buffer at preventing degranulation during the collection step (data not shown). After Ca^2+^ and Mg^2+^ were removed by EGTA and EDTA in the collection step, the cells maintained their intracellular granules. However, only 3% NaCl, in the absence of divalent cations, was used as a washing buffer. This finding indicates that the bleeding solution preparation process can be simplified.

Small-scale experimental data from previous studies revealed that caffeine buffer could inhibit exocytosis. To further confirm this observation, we performed this experiment. Once a week, 6 crabs bled, and the blood of each crab was collected. The results indicated that the caffeine buffer blocks exocytosis during blood collection (Figure 1). The caffeine LAL works in both chromogenic assays and turbidimetric tests. The optimal temperature and pH for the biochemical reaction of caffeine-collected LAL were 37°C and 8.2, respectively, which are slightly different from those of commercial LAL kits, such as the Fungitell recommendation. Interestingly, Ca^2+^ inhibited this reaction (Figure 3B), although it could stimulate exocytosis during the resuspension step (Figure 2D). The mechanism through which caffeine buffer inhibits exocytosis is poorly understood. Our data showed that 50 mM CaCl_2_ could block the effect of caffeine buffer on exocytosis, indicating that Ca^2+^ may be involved in this process, which is consistent with the observation that Ca^2+^ was demonstrated to induce insulin exocytosis in mammalian cells (Eliasson et al., 1997). Additional experiments must be performed to confirm this observation. Most importantly, caffeine buffers are easily prepared. After caffeine and NaCl were completely dissolved in water, the pH of the solution was nearly 6.0, which was almost the optimal pH for the collection buffer.

Notably, endotoxin testing by recombinant factor C (rFC) is currently available. However, this test is more expensive than the traditional *Limulus* amoebocyte lysate test. Moreover, the recombinant factor C (rFC) reagent test works only in chromogenic tests, making some traditional tests, such as gel clots and turbidimetric assays, invalid. Customers of gel clot must purchase new instruments such as plate readers to test endotoxins, increasing their cost. Importantly, recombinant Factor G has not been commercially available until now. Some technical problems need to be solved. Therefore, this caffeine buffer method may be a good method for manufacturing. In particular, the current equipment could be used to produce caffeine lysate and reduce the cost of updating the device.

This study has several limitations. The number of crabs was not sufficient. Middle-scale and large-scale experiments need to be carried out to confirm these observations. Importantly, caffeine enzymes work in both chromogenic assays and turbidimetric tests, and current customers do not need to purchase new instruments for endotoxin tests, which also saves customers’ costs.

In summary, caffeine collection buffers can inhibit exocytosis during blood collection. The use of caffeine buffer is a simple, easily prepared method, and caffeine-collected enzymes work in both chromogenic tests and turbidimetric assays.

## Data Availability

The raw data supporting the conclusions of this article will be made available by the authors, without undue reservation.
